# Molecular epidemiology of swinepox viruses circulating in India

**DOI:** 10.1080/01652176.2022.2150791

**Published:** 2023-01-05

**Authors:** Ashok Kumar, Nikunj Gupta, Arfa Fayaz, Rajangam Mageswary, Rukhsana Bano, Shanmugam ChandraSekar, Dhanavelu Muthuchelvan, Kuldeep Dhama, Awadh B. Pandey, Muthannan Andavar Ramakrishnan

**Affiliations:** a Division of Virology, ICAR-Indian Veterinary Research Institute, Mukteswar, Uttarakhand, India; b Division of Pathology, ICAR-Indian Veterinary Research Institute, Bareilly, Uttar Pradesh, India

**Keywords:** Porcine, pig, swinepox, molecular epidemiology, host range genes, phylogeny, lineage

## Abstract

Swinepox is a sporadic virus disease of domestic and wild pigs that mainly occurs during the rainy season. Though the disease is known for a century, research on swinepox especially genetic characterization is scanty. Self-limiting nature of the disease, the non-availability of specific diagnostics as well as the resemblance of clinical signs with other pathogens are some of the issues in the slow progress in swinepox-related research. Recent whole genome sequencing data from the USA, India, and Germany enhanced our understanding of the biology of swinepox virus (SWPV). The objective of the present study is to investigate the molecular epidemiology of two swinepox outbreaks that occurred in 2015 and 2016 one each in Uttar Pradesh, and the Haryana states of India. The appearance of clinical signs in different swine breeds was recorded. The scab samples from infected pigs were collected, DNA extracted, host range genes of SWPV were PCR amplified, sequenced and analyzed for genetic and phylogenetic characterization. Desi (nondescript breed), Yorkshire White pigs, and Landrace cross were found to be infected with SWPV. Host range genes of SWPV analyzed from clinical samples showed very high nucleotide identity with each other. Phylogenetic analyses revealed that SWPVs circulating in India are distinct (Indian lineage) from the SWPV of the USA, Germany, and Russia (European-North American lineage). Our study affirms the existence of two distinct lineages of SWPV globally with differences in clinical lesions between breeds.

## 1. Introduction

Swinepox virus (SWPV), the causative agent of swinepox disease, has been classified as the only member of the genus *Suipoxvirus* in the subfamily *Chordopoxvirinae* of the *Poxviridae* family (Delhon et al. 2007). Swinepox has been reported in North America, South America, Europe, and India (Ramakrishnan and Ashokkumar 2019) with outbreaks occurring mainly during the rainy season. The primary cause of the spread of the disease is inadequate sanitary conditions where pigs are raised. In addition to direct contact and congenital infection, it is believed that the virus could be transmitted mechanically by pig lice (*Haematopinus suis*) and domestic flies (*Musca domestica*) (Borst et al. 1990; Thibault et al. 1998; Delhon et al. 2007; Mittal et al. 2011; Kaiser et al. 2021). SWPV infection is associated with high morbidity in piglets of 3–4 months old and pigs are the only hosts and reservoirs of this pathogen. SWPV-elicited disease in adults is less pathogenic, self-limiting, and of limited economic impact. Several outbreaks have been reported in Assam, and Haryana states of India (Mittal et al. 2011; Jindal et al. 2015; Riyesh et al. 2016; Mech et al. 2018; Aasdev et al. 2021). Recently, swinepox outbreaks were reported in both domestic and wild pigs in Germany (Kaiser et al. 2021). The condition develops mainly as a skin pustular lesion, including hydropic degeneration of skin stratum spinosum. Swinepox diagnosis can be achieved by histopathology (Cheville 1966a), electron microscopy (Teppema and De Boer 1975; Mittal et al. 2011), immunofluorescence (Cheville 1966b), and PCR (Jindal et al. 2015; Medaglia et al. 2015; Riyesh et al. 2016).

Although the causative agent of swinepox was identified a century ago, the molecular epidemiology of the disease is poorly understood. Very recently only, three whole genome data have been published i.e. in addition to the existing USA SWPV reference sequence (Afonso et al. 2002), one from India (Aasdev et al. 2021) and two from Germany (one each from domestic and wild pig) (Kaiser et al. 2021). SWPV is comprised of a central coding region (∼139 kb) and inverted terminal repeats (∼3.7 kb each). Further, the genome can be demarcated as core or central conserved regions (ORFs 21 to 125) and variable terminal regions (ORFs 1 to 20; and 126 to 150). The genes in the core are mainly involved in the replication process and genes in terminal regions are important in determining host range, immune evasion, virulence, etc. (Afonso et al. 2002; Delhon et al. 2007; Bratke et al. 2013). The name host range genes are mainly related to the tropism of cell culture rather than the host system; the host range genes are dispensable for the permissiveness of cell culture but require growth in animals (McFadden 2005; Haller et al. 2014; Oliveira et al. 2017). About 12 groups of host range genes are identified from different poxviruses but none of them is common for all the genera (Oliveira et al. 2017).

The host range genes of SWPV largely overlap with those found in other clade II poxviruses, but they have a unique gene profile, with the notable absence of orthologs of T4, ANK/F-box group 6, and TNFR-2-related genes found in other clade II poxviruses (Bratke et al. 2013). In comparison with the related LSDV, 13 genes (including the T4 ortholog) are missing in SWPV (Afonso et al. 2002). Work on SWPV host range genes has been very limited and therefore, the present study was aimed to characterize the Indian SWPV at genetic and phylogenetic levels from the two swinepox outbreaks which occurred one each from Haryana and Uttar Pradesh states of India, respectively. The scabs samples from the animal showing pox lesions were collected and genetically and phylogenetically characterized by targeting five host range genes (ORFs 01, 05, 07, 119, and 120) for the Haryana outbreak and two host range genes for the UP outbreak (ORFs 01, and 119).

## 2. Materials and methods

### 2.1. Sample collection and processing

**Figure 1.**
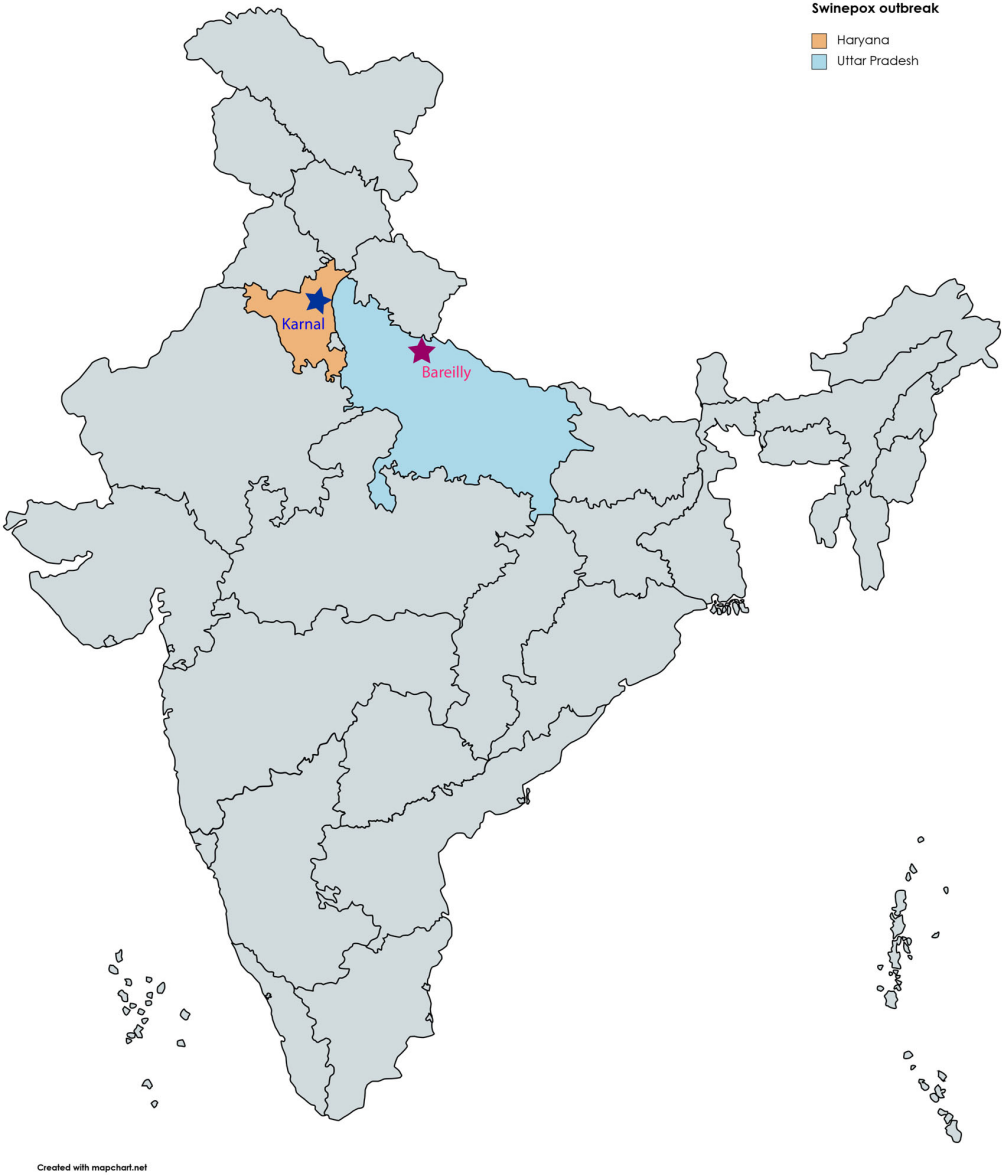
Map showing locations of clinical outbreaks of swinepox that occurred in two Indian states viz., Haryana, and Uttar Pradesh.

In October 2015, there was a suspected swinepox outbreak in commercial pig farms near the town of Nilokheri, Karnal District, in the Indian state of Haryana. A team from the Division of Virology, ICAR-Indian Veterinary Research Institute (IVRI), Mukteswar, Uttarakhand, India visited the affected farms on 6 October 2015. The affected animals in this outbreak belong to Yorkshire White pigs. The team also investigated another outbreak that occurred in April 2016 on a pig farm located in Bareilly District of Uttar Pradesh. In the latter outbreaks, the animals affected were Landrace cross, and Desi (local non-descriptive black) breeds (Table 1). The scab samples were collected and transported to the IVRI in Mukteswar for virological and molecular examinations. The outbreak location map is depicted in Figure 1.

**Table 1.** Details of investigation carried out, farm history and sample collection details.

S. No.	Collection Date	Source of sample	Description	Name assigned to the sample	Remarks
1.	06/10/2015	Private pig farm, Karnal, Haryana	Skin scab	Swinepox virus/swine/India/Haryana/IVRI-M/Karnal-scab1/2015	The outbreak occurred in October 2015. Total pigs availabl*e* = 274 Yorkshire white pigs. Clinical signs were observed in 7 animals and skin scabs were pooled.
2	06/10/2015	Private pig farm, Karnal, Haryana	Skin scab	Swinepox virus/swine/India/Haryana/IVRI-M/Karnal-scab2/2015	The outbreak occurred in October 2015. Total pigs availabl*e* = 40 Yorkshire white pigs. Clinical signs observed in 3animals and skin scabs were pooled
3	06/10/2015	Private pig farm, Karnal, Haryana	Skin scab	Swinepox virus/swine/India/Haryana/IVRI-M/Karnal-scab3/2015	The outbreak occurred in October 2015. Total pigs availabl*e* = 31 Yorkshire white pigs. Clinical signs were observed in 3 animals and skin scabs were pooled
4	17/04/2016	Institutional swine farm, Izatnagar, U.P.	Skin scab	Swinepox-virus/swine/India/Izatnagar /U.P./IVRI-M/IZT-scab1/2016	The outbreak occurred in April 2016. Sample Nos. 4, 5, and 6 were collected from the same farm. Total animals availabl*e* = 46 (Landrace cros*s* = 17 female and 26 male; Des*i* = 3 females) Sample No. 4: Pooled skin scabs from female Landrace cross (*n* = 5) Sample No. 5: Pooled skin scabs from male Landrace cross (*n* = 5) Sample No. 6: Pooled skin scabs from female Desi (*n* = 3)
5	17/04/2016	Institutional swine farm, Izatnagar, U.P.	Skin scab	Swinepox virus/swine/India/Izatnagar/U.P./C.B.-M, SWPV-IZT--scab 2-2016	
6	17/04/2016	Institutional swine farm, Izatnagar U.P.	Skin scab	Swinepox virus/swine/India/Izatnagar/U.P./C.B.-F, SWPV-IZT--scab 3-2016	

### 2.2. Method

The primers used for the PCR and sequencing of SWPV genes are listed in Table 2. The primers were designed using primer 3 PLUS software with reference sequences available in GenBank (NC_003389.1). Clinical samples were homogenized in phosphate-buffered saline (PBS, pH 7.2) to generate a 10% suspension and then treated with a double dose of Antibiotic Antimycotic Solution (Himedia, India). DNA from clinical samples was extracted using a modified FNES (fast, non-enzymatic, and simple) protocol described previously (Santhamani et al. 2013). The quantity and purity of the DNA obtained by this method were determined using a NanoDrop spectrophotometer.

**Table 2.** Details of primers and GenBank accession number for the sequences submitted in the present study.

Target ORF (Function)	Primer name	Sequence (5’ – 3’)	**Primer** **length (base)**	**Amplicon** **size (bp)**	**PCR Annealing** **Temp (°C)**	**GenBank Submission.** **(Accession no for the present study)**
ORF-001 (A52R family protein)	SWPV-1-175-F	aaaaacaaacgcatactttttga	23	691	64.7	MG792013, MG792014, MG792015, MG874784, MG874785, MG874786
	SWPV-1-865-R	gattccaaatccttttcgtaaata	24			
ORF-005 (GPCR, TM)	SWPV-5-2608-F	actatccagcgacagccaac	20	1074	55.8	MG792016, MG792017 MG792018
	SWPV5-3681-R	gcgttcaaaagaaacaaatcc	21			
ORF-007 (A52R family protein)	SWPV7-5181-F	cctatgtgcgctaatggtttt	21	794	46.4	MG843847, MG843848 MG843849
	SWPV-7-6155-R	ccaatatatcgtcaaagtttatgcac	26			
ORF-119 (EEV glycoprotein, TM)	SWPV-119-111438-F	caaatacatagttttgccagatcc	24	909	64.7	MG874778, MG874779 MG874780, MG931947 MG931948, MG931949
	SWPV-119-112346-R	agctgcgggtgttgttaatc	20			
ORF-120 (EEV protein)	SWPV-120-112162-F	tggggtgaagaaggtagagga	21	739	63.1	MG874781, MG874782 MG874783
	SWPV-120-112900-R	aacgatcttgatacattatcttctgat	27			

For the genetic characterization studies, five genes (ORFs 01/150, 05/149, 07, 119, and 120) were targeted for the Haryana outbreak and two host range genes (ORFs 001, and 119) for the UP outbreak. The PCR was carried out using Terra™ PCR Direct Red Dye Premix (TAKARA, Mountain View, CA, USA) in a total volume of 25 μL, and the thermal conditions were as follows: initial denaturation at 98 °C for 2 min followed by 35 cycles of 98 °C for 10 s, 30 s and 68.0 °C for 1 min, with a final extension at 68.0 °C for 5 min; the annealing temperatures for the different PCRs are provided in Table 2. To obtain the sequence for a full length in addition to the sequencing of PCR amplicons directly (gel purified), the PCR products were also sequenced by cloning protocol using the pTZ57R/T vector. After confirmation by the gene-specific PCRs, the cultures containing the positive clones (at least two clones per sample) were prepared as stab cultures and sequenced commercially (SciGenom, Cochin, Kerala, India).

### 2.3. Data analysis

Representative nucleotides and deduced amino acid sequences of SWPV were downloaded from the NCBI database and used for comparative analysis. Swinepox Reference Sequence NC003389.1 (GenBank AF410153.1) was used to indicate the positions of nucleotide and amino acid. The multiple alignment and phylogenetic analyses were carried out in MEGA XI software. The phylogenetic tree was constructed using the Neighbor-Joining method with 1000 replicates (Bootstrap method). The evolutionary distances were computed using the K2P method (number of base substitutions per site). The rate variation among sites was modelled with a gamma distribution (shape parameter = 1). All ambiguous positions were removed for each sequence pair i.e. pairwise deletion option (Tamura et al. 2021).

## 3. Results

**Figure 2.**
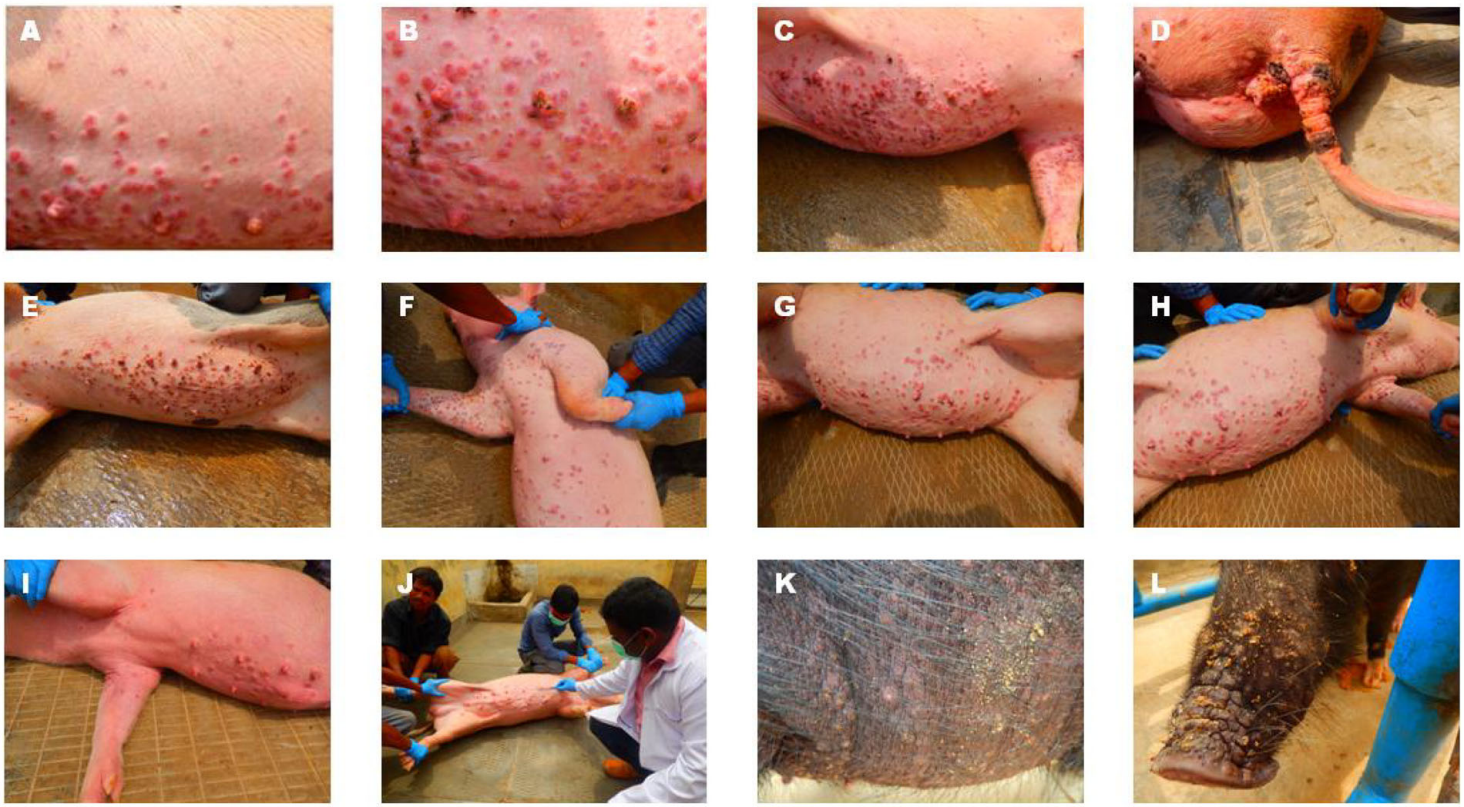
Swinepox lesions in Uttar Pradesh Outbreak. A-J: Landrace Cross; K and L: Desi black (nondescript) breed.

**Figure 3.**
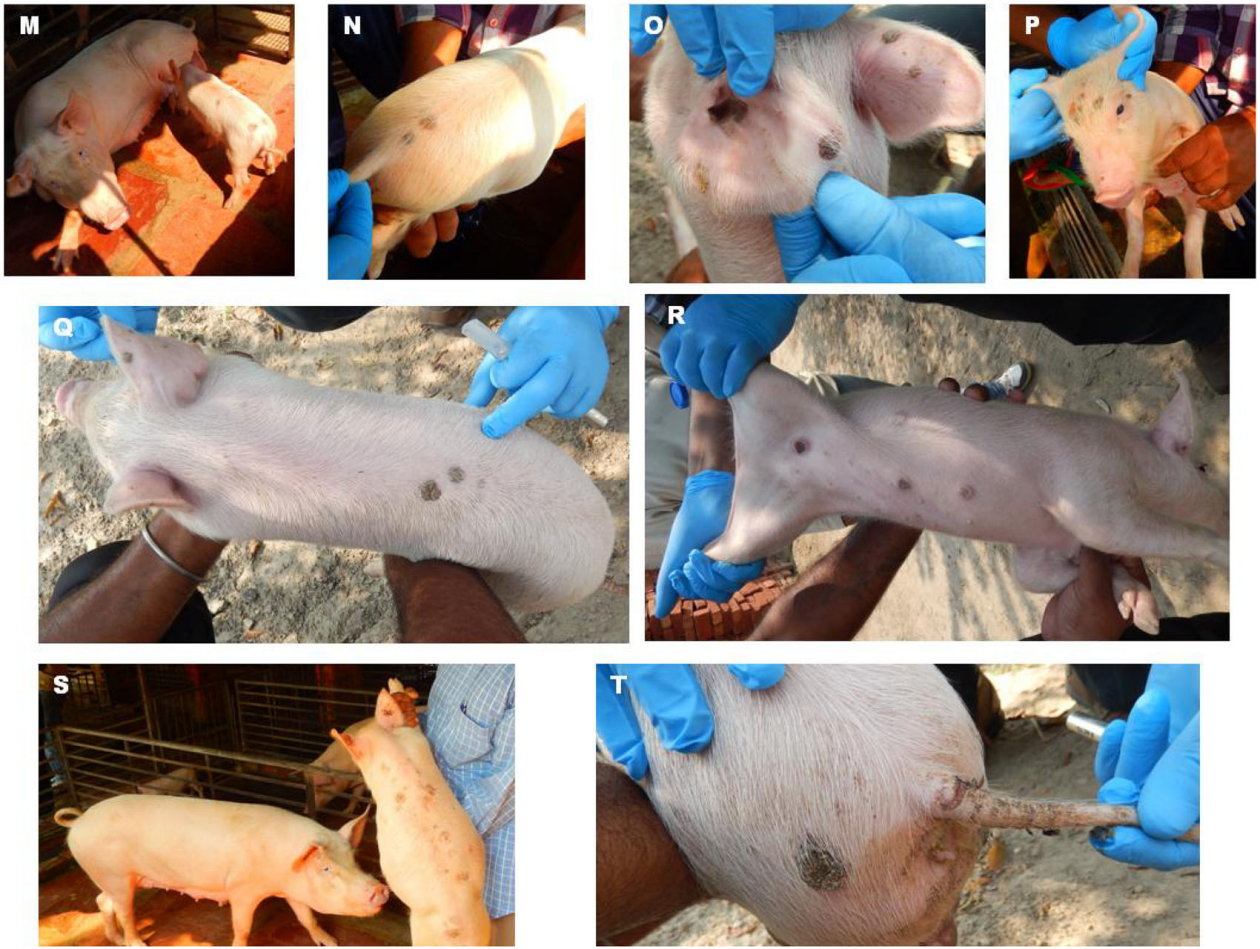
Swinepox lesions (M-T) observed in Yorkshire white pigs in Haryana state.

In the present study, investigations were carried out from two farms one each from UP, and Haryana States. In the UP outbreak, both Landrace crossbred and Desi black (nondescript) breeds. Extensive pox lesions spread over the entire body in Landrace cross and both male and female animals were infected. Though adult animals are considered resistant to infection, severe lesions could be observed in both young and adult animals. Due to the hair and colour of the Desi breeds (black), the visibility of the lesions was low; however, lesions could be observed in the snout, leg and abdominal area (Figure 2). In the Haryana outbreak (Yorkshire white pigs), the severity was comparatively low and lesions appeared larger but with few papules; both male and female young animals showed the lesions but not in adult animals (Figure 3).

The samples collected from Haryana were screened with five host range genes (ORFs 01, 05, 07, 119, and 120) and two host range genes (ORFs 001, and 119) for samples from UP. The category of host range gene/functions is provided in Table 2. The comparative nucleotide and deduced amino acids analyses showed several single nucleotide polymorphisms (SNPs), and a few indels (deletion, and insertion) between the European-North American lineage and Indian lineage. The results of all five ORFs are presented in Table 3; graphical visualization is depicted in Figures S1 to S10 (Supplementary material) and the results are briefly discussed below.

**Table 3.** Summary of differential nucleotide (SNPs, indel) and amino acid markers observed between SWPV of Indian and European-North American lineages.

S. No.	ORF (Gene)	Nucleotide markers (Single nucleotide polymorphism, insertion or deletion indel)	Amino acid markers
**1**	**ORF001/ORF150** **A52R-like family protein**	**18 markers** A36T, C56T, T88G, C100T, A101G, G114A, T189C, G226T, T228G, A272G, T291C, T297C, T319G, C367T, G424A, T429C, G448T, A453T **(this is a stop codon for western isolates whereas frameshift in Indian isolates leads to the extension of 9 additional nts (AGGGATTAA)**. **Therefore, the length of this gene is 453 for western and 462 for Indian SWPV sequences.**	**11 markers** L12F, T19M, L30V, H34C, A76S, K88R, Y91C, S107A, A142T, G150C. A total of 150 aa in European-American isolates whereas **3 additional aa at the terminal (YRD) in Indian SWPV due to frameshift in the expected stop codon.**
**2**	**ORF005/ORF146** **G protein-coupled CC chemokine receptor-like protein"**	**20 markers** A6G, C171A, C193T, C228T, **insertional indel of 3 nts between 309 and 310 (AAT) in Indian SWPV**, C333T, A335G, G340A, G352A, G369A, C472A, G504T, G508A, T510C, A649G, T675C, G706T, G756T, G761A, A780T. **The length of ORF005 in western and Indian SWPV is 453 and 462 nts, respectively.**	**10 markers** P65S, **1 aa insertion between 103 and 104 in Indian SWPV (N)**, E112G, D114N, A118T, M168I, V170I, I217V, V236F, R254K,
**3**	**ORF-007** **(A52R family protein)**	**17 markers** T108C, C241G, G258A, T286G, A397C, C411T, G421T, A423G, T426C, C447T, A453G, T456C, A480G, G510A, A530G, C615A, A621G. The length of ORF007 for both western and Indian SWPV is 711 nts.	**5 markers** P81A, Y96D, A141S, I160M, N177S.
**4**		**12 markers** G46A, C107A, C147T, T150C, C152T, G192A, G237A, G271T, C299A, T355C, G408A, T541G. The length of ORF119 for both western and Indian SWPV is 518 nts.	**6 markers** A16T, S36Y, T51I, A91S, T100K, L181V.
**5**	**ORF-119** **(EEV glycoprotein, TM)**	**14 markers** G38A, G261A, C264T, A285G, G315A, G395A, **3 nts insertional indel between 396 and 397 in Indian isolates (AAT),** T399C. G401A, T405C, T432G, G447A, G453A, T468C. The length of ORF005 in western and Indian SWPVs is **510 and 513 nts, respectively.**	**4 markers** R13K, R132K,**1 aa insertion between 132 and 133 (N) in Indian SWPV**, S134N.

The nucleotide and amino acid positions are based on the genes of the reference sequence (NC_003389.1; AF410153.1:2821-3630-17077-99/Nebraska/USA).

The letter before and after the nt and aa positions indicates the differentiation markers of India and European-North American lineages of SWPV.

ORF001 (ORF 150 is an identical copy) encodes an A52R family protein (Delhon et al. 2012) that likely functions in modulation or evasion of host immune responses, modulation or inhibition of host cell apoptosis, or cell or tissue tropism (Massung et al. 1993; Tulman et al. 2002). The length of the ORF001 for European-North American lineage is 453 nts with a coding capacity of 150 amino acids, while the Indian SWPV sequences had an extension of nine additional nts due to the frameshift in the expected stop codon which resulted in a length of 462 nts with 153 deduced amino acids. Differentiation markers could be observed between these two lineages at both nucleotide and amino acids levels. A total of 18 nts (17 single nucleotide polymorphisms – SNPs and a stretch of 9 nts insertional indel) lead to 11 amino acid differentiation markers (Table 3; Figures S1 and S2). The ORF001 sequence also showed significant homology with other capripoxviruses (data not shown) and caution is required when developing diagnostic assays based on the ORF001 gene or recombinant protein.

Another host range gene targeted was ORF005 (*C3L* gene; ORF 146 is an identical copy) which encodes a 269 amino acid polypeptide (G protein-coupled CC-chemokine-like protein GPCR) with a putative molecular weight of 31.5 kDa. The length of ORF005 of the European-North American lineage was 810 nts, while the Indian lineage had 813nts. This difference in size was due to the insertional indel of three nucleotides (AAT) between the nts 309 and 310. Therefore, the Indian lineage is expected to code for 270 amino acids. Overall, 20 differentiation markers (19 SNPs, and 1 insertional indel) at nt level and 10 markers at aa level have been observed between these two lineages (Table 3 and Figures S3 and S4).

**Figure 4.**
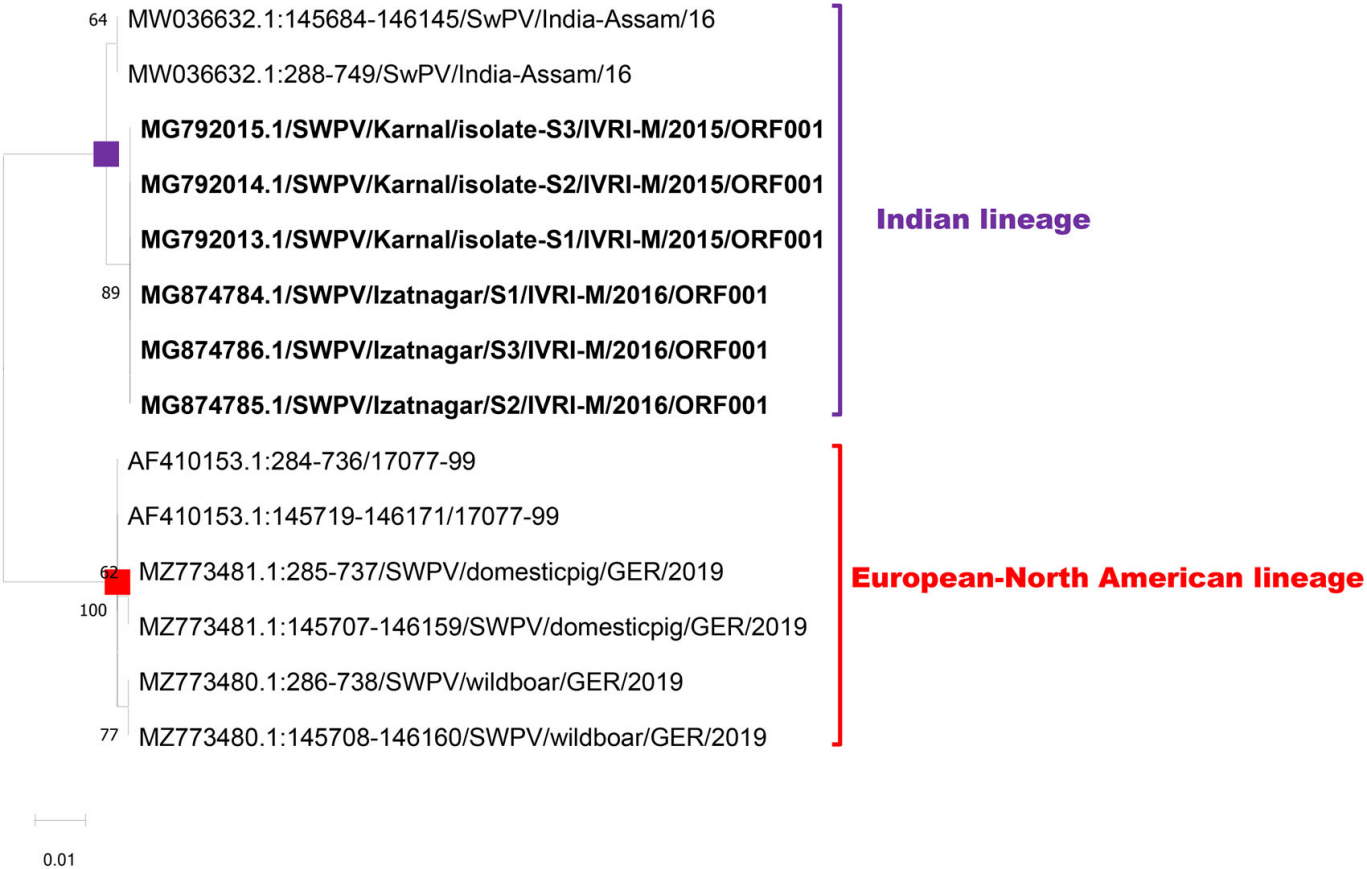
Evolutionary relationships of ORF 001/ORF150 of Indian SWPV with the USA, and Germany SWPV isolates. The isolates of the current study are shown in bold letters.

The third targeted host range gene was ORF007 which encodes the A52R family protein (Alcamí and Smith, 1992). Both the lineages had an ORF length of 711 bp with a coding capacity of 236 amino acids. Overall, differentiation markers were observed in 17, and 5 locations at nt and aa levels, respectively (Table 3; Figures S5 and S6).

**Figure 5.**
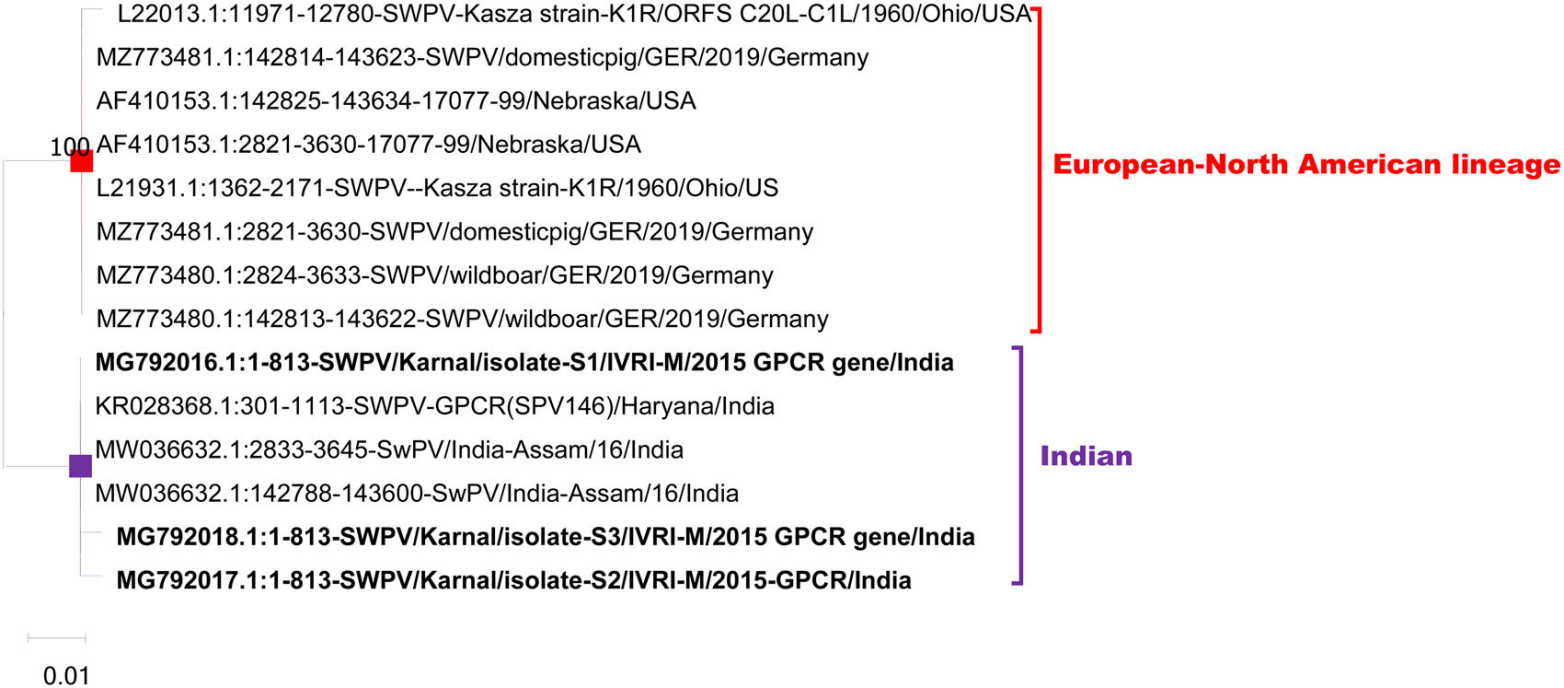
Evolutionary relationships of ORF 005/ORF146 of Indian SWPV with the USA, and Germany SWPV isolates. The isolates of the current study are shown in bold letters.

**Figure 6.**
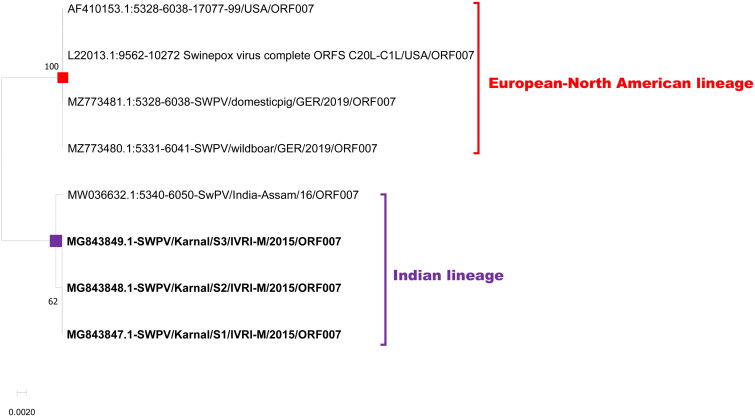
Evolutionary relationships of ORF 007 of Indian SWPV with the USA, and Germany SWPV isolates. The isolates of the current study are shown in bold letters.

ORF119 encodes a polypeptide of 185 amino acids (extracellular enveloped virus proteins - EEV glycoprotein). The expected length of the gene is 558 nts with a coding capacity of 185 amino acids and both the lineages showed the same length and coding capacity. There were, 12 nts and 6 aa differentiation markers observed between the two lineages (Table 3; Figures S7 and S8).

**Figure 7.**
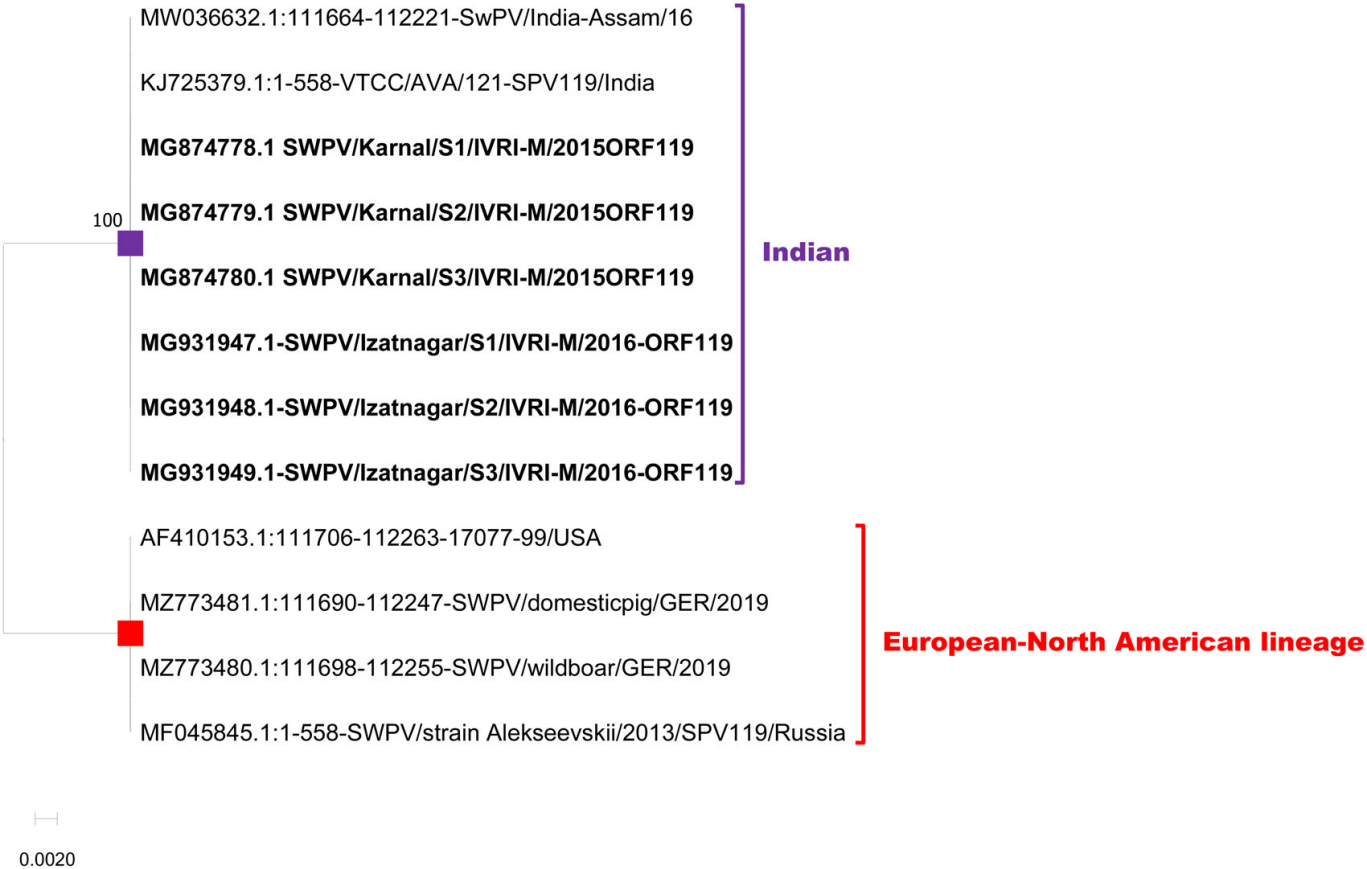
Evolutionary relationships of ORF 119 of Indian SWPV with the USA, and Germany SWPV isolates. The isolates of the current study are shown in bold letters.

**Figure 8.**
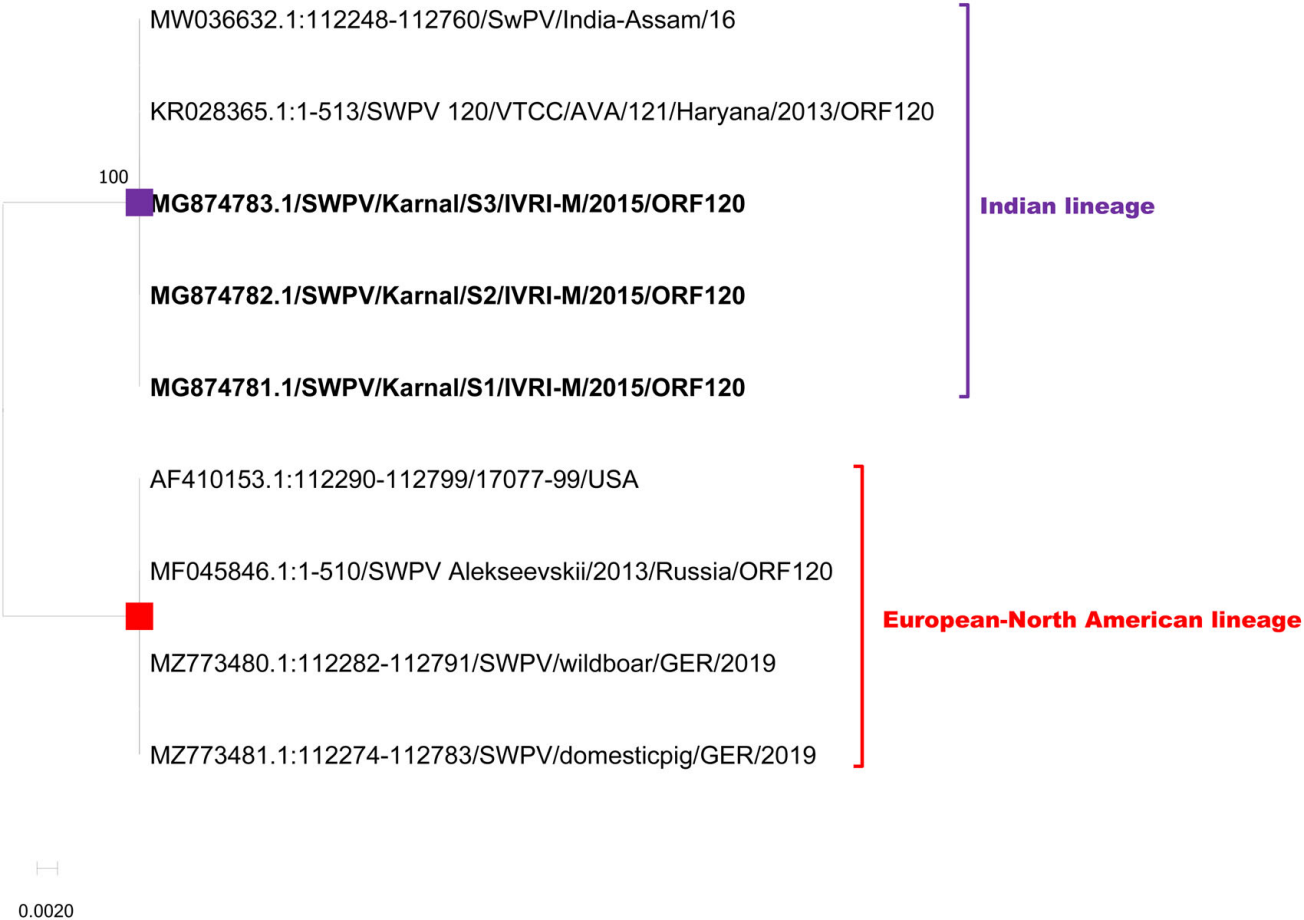
Evolutionary relationships of ORF ORF120 of Indian SWPV with the USA, and Germany SWPV isolates. The isolates of the current study are shown in bold letters.

ORF120 encodes a polypeptide of 169 amino acids (extracellular enveloped virus protein) which is associated with virulence (Massung et al. 1993). The length of the gene of the European-North American lineage is 510 nts which encodes 169 amino acids. However, the Indian lineage had 513 nts, with a coding capacity of 170 amino acids. This size difference is due to the insertional indel of three nucleotides (AAT) between positions 396 and 397 (N). Overall, there were 14 nts and 4 aa differentiation markers observed between the two lineages (Table 3; Figures S9 and S10).

The BLAST analyses of the above five genes reveal that all the Indian SWPV (Indian lineage) shared more than 99% nts identity within them. Similarly European and North American SWPV (European-North American lineage) shared >99% identity. However, there was a significant divergence between SWPV of India and European-North American lineage. It was observed that 95.39%, 96.92%, 97.47%, 97.85%, and 97.08% nts identity for ORFs 1, 5, 7, 119, and 120, respectively which correlated with the SNPs and indels observed between the Indian and western SWPV sequences.

The phylogenetic tree with all the five genes used in the current study deciphered that there are two distinct clades of SWPV viz., the Indian SWPV lineage and the European-North American SWPV lineage (Figures 4–8). The latter clade includes sequences from USA, German, and Russia (available for a few genes only).

## 4. Discussion

The present investigation reveals that all breeds of pigs are susceptible though the severity, size and type of cutaneous lesions may vary. In the previous studies also multiple cutaneous lesions were observed from different parts of the body (McNutt et al. 1929; De Boer 1975; Kim and Luong 1975; Olufemi et al. 1981; Jubb et al. 1992). In congenital infections, lesions may appear on the entire body and in the case of mechanical transmission by the vector, the lesions can be found in the feeding areas of the vector (Delhon et al. 2007). Further, it was reported that both domestic and wild pigs can be infected by SWPV. House flies are one of the possible mechanical vectors, and the presence of these flies is presented on both farms. Therefore, is difficult to conclude the source of infections. In a recent swinepox outbreak in Germany, the farms practiced all the hygienic measures and therefore mechanical vector transmission is unlikely; the transmission might have occurred through persistently infected asymptomatic animals (Kaiser et al. 2021).

After the first complete SWPV genome was published in 2002 (Afonso et al. 2002), a few months ago only, three additional sequences were published viz., one from India (domestic pig) (Aasdev et al. 2021) and two from Germany (one each from domestic and wild pigs) (Kaiser et al. 2021). In addition, a few host range genes of SWPV are also characterized by Indian researchers (Riyesh et al. 2016). The availability of sequence data enhanced our understanding of the biology and epidemiology of SWPV from different countries.

The DNA was extracted using the modified FNES protocol (Santhamani et al. 2013). The concentration obtained was ∼25 ng/μL per sample and the 260/280 ratio was above 1.8, indicating that the DNA had a high degree of purity. This protocol has been already employed for other pox viruses like goatpox and sheeppox (Ramakrishnan et al. 2017) now we have incorporated it successfully for the swinepox virus also.

Recent three studies enhanced our knowledge of the evolutionary relationship of SWPV from different countries. In one study, analyses of host range genes of SWPV (Riyesh et al. 2016) reveals that Indian SWPVs are genetically closely related to SWPV of the USA. In 2021, the first whole genome sequence of Indian SWPV was carried out and it was opined that the Indian isolate belongs to Eurasian lineage and diverges significantly from American lineage (Aasdev et al. 2021). Almost in the same period, interesting data were obtained from the whole genome sequencing of SWPV from domestic and wild pigs by researchers from Germany (Kaiser et al. 2021). Their study indicates that the USA and German isolates clustered under one lineage whereas Indian SWPV formed a separate lineage; further differences could be observed between some of the ORFs of domestic and wild pig origin (Bohórquez et al. 2021). It was hypothesized that the two lineages of SWPV could have evolved at a certain point of time during the domestication of pigs (Aasdev et al. 2021; Kaiser et al. 2021). Our study affirms that Indian SWPVs are distinct from European-North American lineage. Need more data to understand either lineage-level classification is sufficient or need to classify them into species levels. In the *Genus Capripoxvirus,* there are three species viz., sheeppox, goatpox, and LSDV of cattle were recognized based on the sequence-specific markers in some genes and/or the entire genome (Ramakrishnan et al. 2017). The comparative pathogenicity, immunogenicity, and vaccine efficacy of both lineages will shed a plethora of information on SWPV.

In conclusion, all five host range genes of the Indian SWPV sequences showed significant divergence from the SWPVs of European- North American SWPV sequences due to the high variations at both the nucleotide and amino acid levels. Although the two outbreaks occurred several miles apart, the molecular epidemiological studies indicate that the Indian SWPV might have originated from a single gene pool. The findings of the present study will help in better understanding of the molecular epidemiology of the SWPV circulating in India and aid in the development of vaccines and nucleo-diagnostic tools.

## Supplementary Material

Supplemental Material

## References

[CIT0001] Aasdev A, Mishra A, Bora DP, Kurkure NV, Barman NN, Raut AA. 2021. First complete genome characterization of swinepox virus directly from a clinical sample indicates divergence of a Eurasian-lineage virus. Arch Virol. 166(4):1217–1225. 33550505 10.1007/s00705-021-04971-w

[CIT0002] Afonso CL, Tulman ER, Lu Z, Zsak L, Osorio FA, Balinsky C, Kutish GF, Rock DL. 2002. The genome of swinepox virus. J Virol. 76(2):783–790. 11752168 10.1128/JVI.76.2.783-790.2002 PMC136851

[CIT0003] Alcamí A, Smith GL. 1992. A soluble receptor for interleukin-1 beta encoded by vaccinia virus: a novel mechanism of virus modulation of the host response to infection. Cell. 71(1):153–167. 1394428 10.1016/0092-8674(92)90274-g

[CIT0004] Bohórquez JA, Defaus S, Rosell R, Pérez-Simó M, Alberch M, Gladue DP, Borca MV, Andreu D, Ganges L. 2021. Development of a dendrimeric peptide-based approach for the differentiation of animals vaccinated with FlagT4G against classical swine fever from infected pigs. Viruses. 13(10):1980. 34696410 10.3390/v13101980 PMC8540558

[CIT0006] Borst GH, Kimman TG, Gielkens AL, van der Kamp JS. 1990. Four sporadic cases of congenital swinepox. Vet Rec. 127(3):61–63. PMID: 2169139 2169139

[CIT0007] Bratke KA, McLysaght A, Rothenburg S. 2013. A survey of host range genes in poxvirus genomes. Infect Genet Evol. 14:406–425. 23268114 10.1016/j.meegid.2012.12.002 PMC4080715

[CIT0009] Cheville NF. 1966a. Immunofluorescent and morphologic studies on swinepox. Pathol Vet. 3(5):556–564. 4289425 10.1177/030098586600300512

[CIT0010] Cheville NF. 1966b. The cytopathology of swine pox in the skin of swine. Am J Pathol. 49(2):339–352. PMID: 4287838 4287838 PMC1907237

[CIT0012] De Boer GF. 1975. Swinepox. Virus isolation, experimental infections and the differentiation from vaccinia virus infections. Arch Virol. 49(2-3):141–150. 174522 10.1007/BF01317533

[CIT0013] Delhon G, Tulman ER, Afonso CL, Rock DL. 2012. Diseases of swine. 10th ed. Chichester, West Sussex; Ames, Iowa: Wiley-Blackwell.

[CIT0014] Delhon GA, Tulman ER, Afonso CL, Rock DL. 2007. Genus Suipoxvirus. In: Mercer AA, Schmidt A, Weber OF, editors, Poxviruses, Birkhäuser advances in infectious diseases. Basel, Boston: Birkhäuser; p. 203–215.

[CIT0015] Haller SL, Peng C, McFadden G, Rothenburg S. 2014. Poxviruses and the evolution of host range and virulence. Infect Genet Evol. 21:15–40. 24161410 10.1016/j.meegid.2013.10.014 PMC3945082

[CIT0016] Jindal N, Barua S, Riyesh T, Lather A, Narang G. 2015. Molecular detection of swinepox virus in two piggery units in Haryana state. Haryana Vet. 54:72–74.

[CIT0017] Jubb TF, Ellis TM, Peet RL, Parkinson J. 1992. Swinepox in pigs in northern Western Australia. Aust Vet J. 69(4):99. 1318716 10.1111/j.1751-0813.1992.tb15566.x

[CIT0018] Kaiser FK, Wiedemann A, Kühl B, Menke L, Beineke A, Baumgärtner W, Wohlsein P, Rigbers K, Becher P, Peters M, et al. 2021. Swinepox virus strains isolated from domestic pigs and wild boar in Germany display altered coding capacity in the terminal genome region encoding for species-specific genes. Viruses. 13(10):2038. 34696467 10.3390/v13102038 PMC8538704

[CIT0021] Kim JCS, Luong LC. 1975. Ultrastructure of swine pox. Vet Med Small Anim Clin. 70(9):1043–1045. PMID: 170726 170726

[CIT0022] Massung RF, Jayarama V, Moyer RW. 1993. DNA sequence analysis of conserved and unique regions of swinepox virus: identification of genetic elements supporting phenotypic observations including a novel G protein-coupled receptor homologue. Virology. 197(2):511–528. 8249275 10.1006/viro.1993.1625

[CIT0023] Mech P, Bora DP, Neher S, Barman NN, Borah P, Tamuly S, Dutta LJ, Das SK. 2018. Identification of swinepox virus from natural outbreaks in pig population of Assam. Virus Dis. 29(3):395–399. 10.1007/s13337-018-0464-2 PMC6111949 30159378

[CIT0024] McFadden G. 2005. Poxvirus tropism. Nat Rev Microbiol. 3(3):201–213. 15738948 10.1038/nrmicro1099 PMC4382915

[CIT0025] McNutt SH, Murray C, Purwin P. 1929. Swine pox. J Am Vet Med Assoc. 74:752–761.

[CIT0027] Medaglia MLG, Sá NMB, Correa IA, Costa LJ, Damaso CR. 2015. One-step duplex polymerase chain reaction for the detection of swinepox and vaccinia viruses in skin lesions of swine with poxvirus-related disease. J Virol Methods. 219:10–13. 25804245 10.1016/j.jviromet.2015.03.010

[CIT0028] Mittal D, Mahajan V, Pathak D, Filia G. 2011. Differential diagnosis of swine pox during an outbreak. Indian Vet. J. 88:9–11.

[CIT0029] Oliveira GP, Rodrigues RAL, Lima MT, Drumond BP, Abrahão JS. 2017. Poxvirus host range genes and virus–host spectrum: a critical review. Viruses. 9(11):331. 29112165 10.3390/v9110331 PMC5707538

[CIT0030] Olufemi BE, Ayoade GO, Ikede BO, Akpavie SO, Nwufoh KJ. 1981. Swine pox in Nigeria. Vet Rec. 109(13):278–280. 6278693 10.1136/vr.109.13.278

[CIT0031] Ramakrishnan MA, Ashokkumar D. 2019. Swinepox virus. In Recent advances in animal virology. Singapore: Springer; p. 161–169.

[CIT0032] Ramakrishnan MA, Santhamani R, Pandey AB. 2017. Capripox outbreak in a mixed flock of sheep and goats in India. Transbound Emerg Dis. 64(1):27–30. 28028940 10.1111/tbed.12604

[CIT0033] Riyesh T, Barua S, Kumar N, Jindal N, Bera BC, Narang G, Mahajan NK, Arora D, Anand T, Vaid RK, et al. 2016. Isolation and genetic characterization of swinepox virus from pigs in India. Comp Immunol Microbiol Infect Dis. 46:60–65. 27260812 10.1016/j.cimid.2016.04.001

[CIT0035] Santhamani R, Yogisharadhya R, Venkatesan G, Shivachandra SB, Pandey AB, Ramakrishnan MA. 2013. Detection and differentiation of sheeppox virus and goatpox virus from clinical samples using 30kDa RNA polymerase subunit (RPO30) gene based PCR. Vet World. 6(11):923–925.

[CIT0037] Tamura K, Stecher G, Kumar S. 2021. MEGA11: molecular evolutionary genetics analysis version 11. Mol Biol Evol. 38(7):3022–3027. 33892491 10.1093/molbev/msab120 PMC8233496

[CIT0038] Teppema JS, De Boer GF. 1975. Ultrastructural aspects of experimental swinepox with special reference to inclusion bodies. Arch Virol. 49(2-3):151–163. 174523 10.1007/BF01317534

[CIT0039] Thibault S, Drolet R, Alain R, Dea S. 1998. Congenital swine pox: a sporadic skin disorder in nursing piglets. Swine Health Prod. 6:276–278.

[CIT0041] Tulman ER, Afonso CL, Lu Z, Zsak L, Sur J-H, Sandybaev NT, Kerembekova UZ, Zaitsev VL, Kutish GF, Rock DL. 2002. The genomes of sheeppox and goatpox viruses. J Virol. 76(12):6054–6061. 12021338 10.1128/JVI.76.12.6054-6061.2002 PMC136203

