# Supplementary material for: Molecular epidemiology of swinepox viruses circulating in India
Source: Vet Q. 2023 Jan 5;43(1):1–10. doi: 10.1080/01652176.2022.2150791 (PMC9828724; doi:10.1080/01652176.2022.2150791)
Supplement: Supplemental Material [file TVEQ_A_2150791_SM2161.docx]

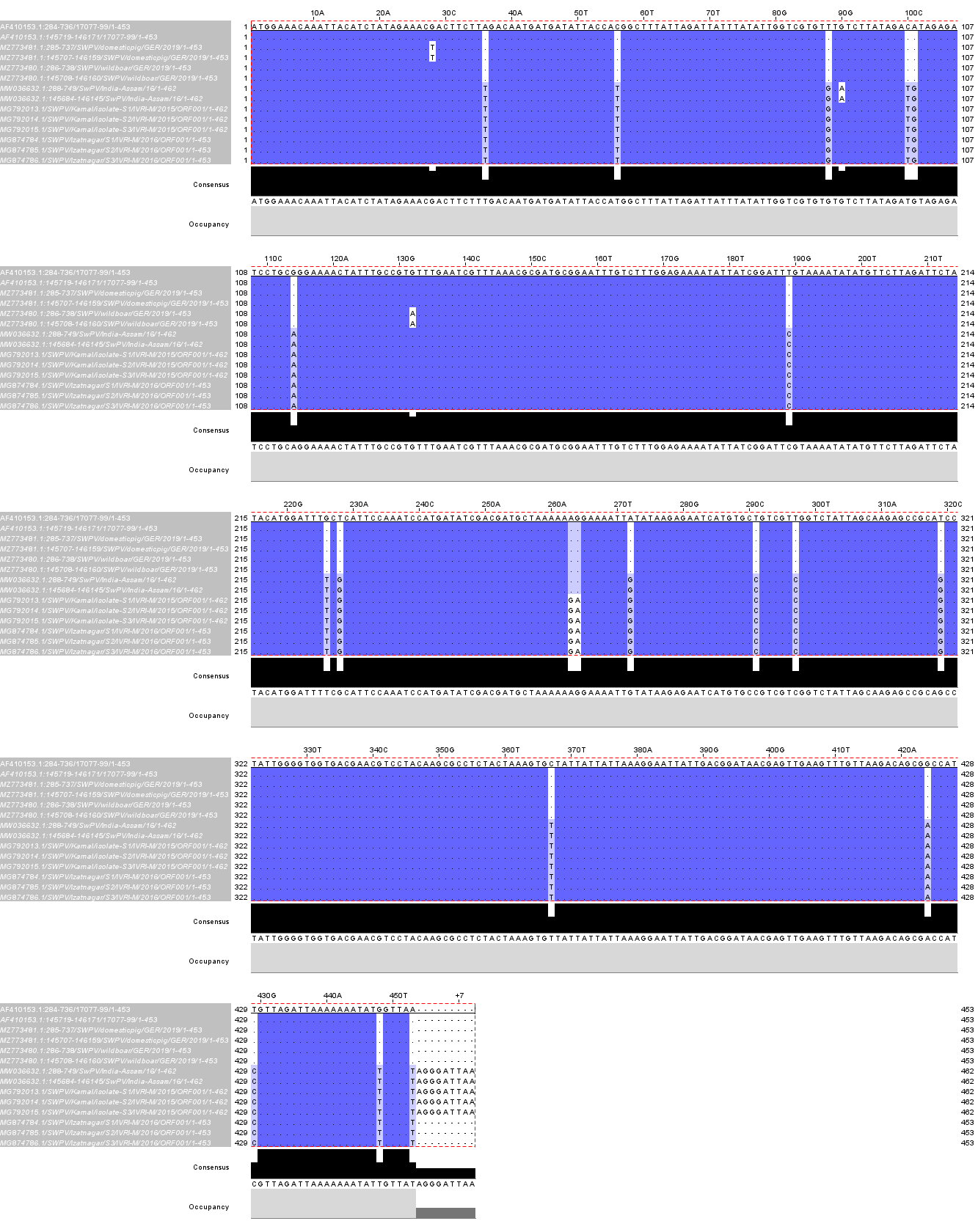


Fig. S1. Visualization of differential nucleotide markers (indels, SNPs) in ORF001/ORF150 of SWPV using Jalview 2.11.2.3. The conserved nts are dotted on the blue background and the differential markers are shown on the white background. A total of 18 differential markers could be observed between Indian lineage and European-North American lineage. Some isolate-specific markers could also be observed.


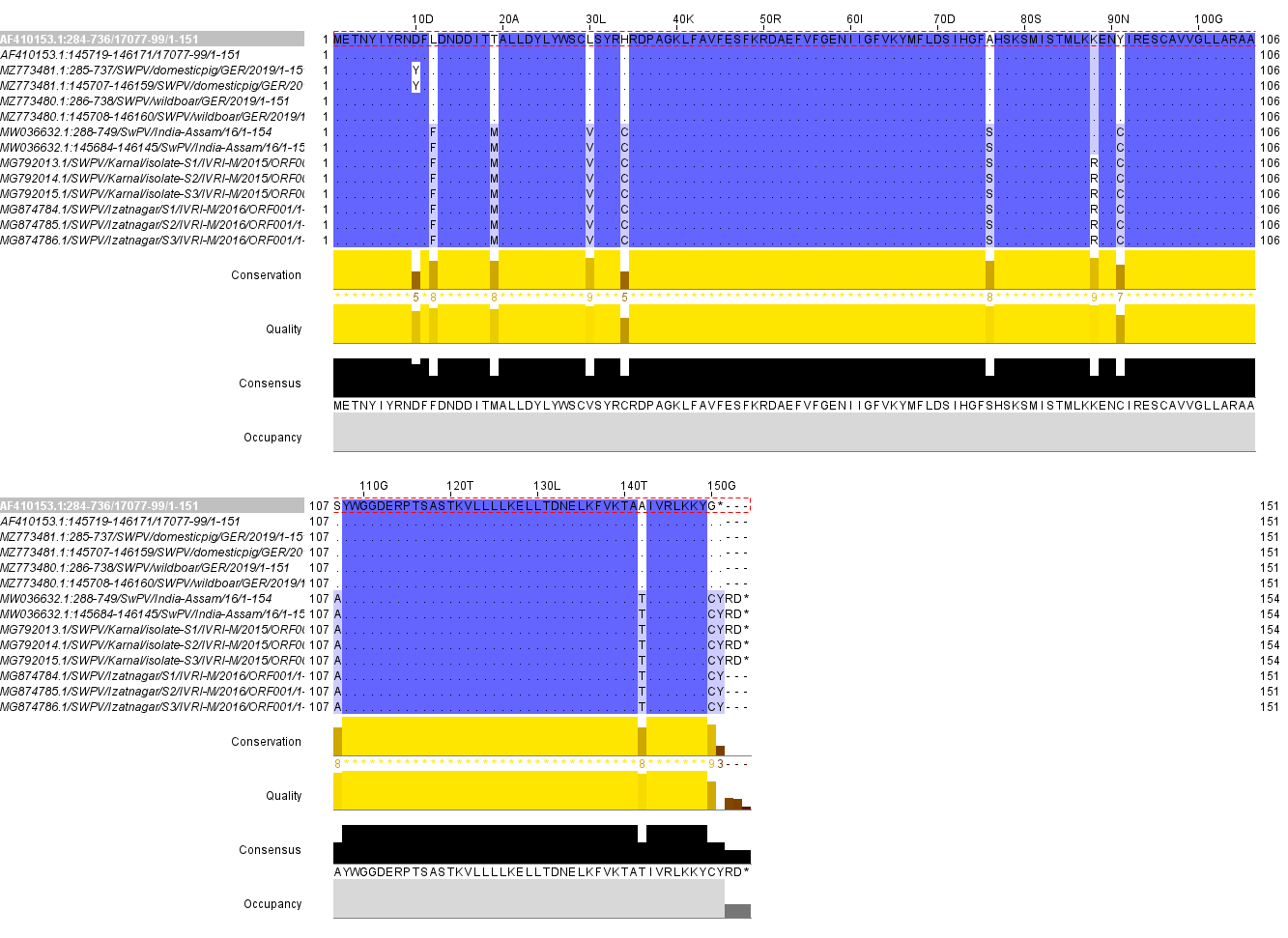


Fig. S2. Visualization of differential amino acid markers (indels, SNPs) in ORF001/ORF150 of SWPV using Jalview 2.11.2.3. The conserved nts are dotted on the blue background and the differential markers are shown on a white background. A total of 11 differential markers could be observed between Indian lineage and European-North American lineage. Some isolate-specific markers could also be observed.


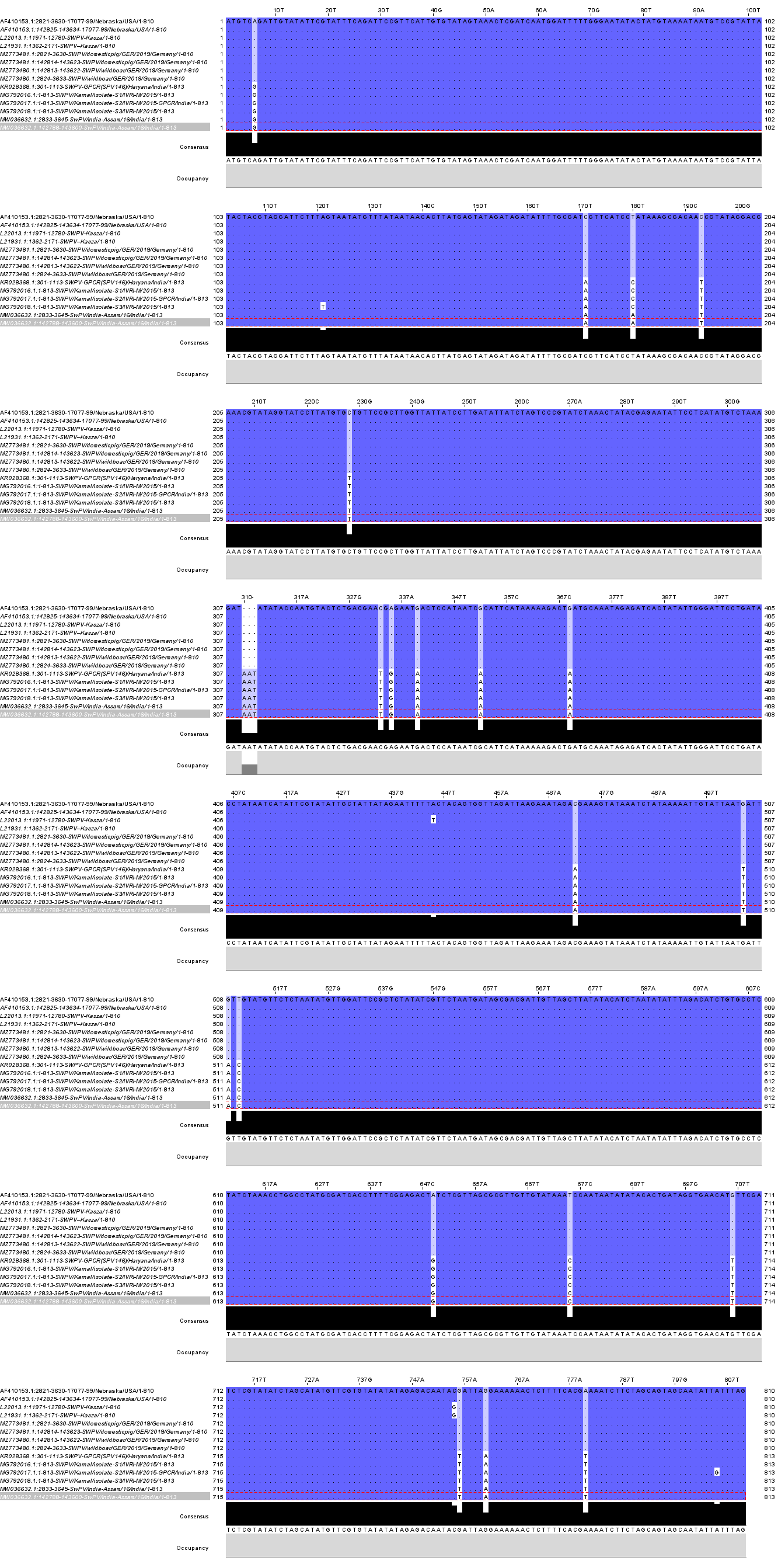


Fig. S3. Visualization of differential nucleotide markers (indels, SNPs) in ORF005/ORF146 of SWPV using Jalview 2.11.2.3. The conserved nts are dotted on the blue background and the differential markers are shown on the white background. A total of 20 differential markers could be observed between Indian lineage and European-North American lineage. Some isolate-specific markers could also be observed.


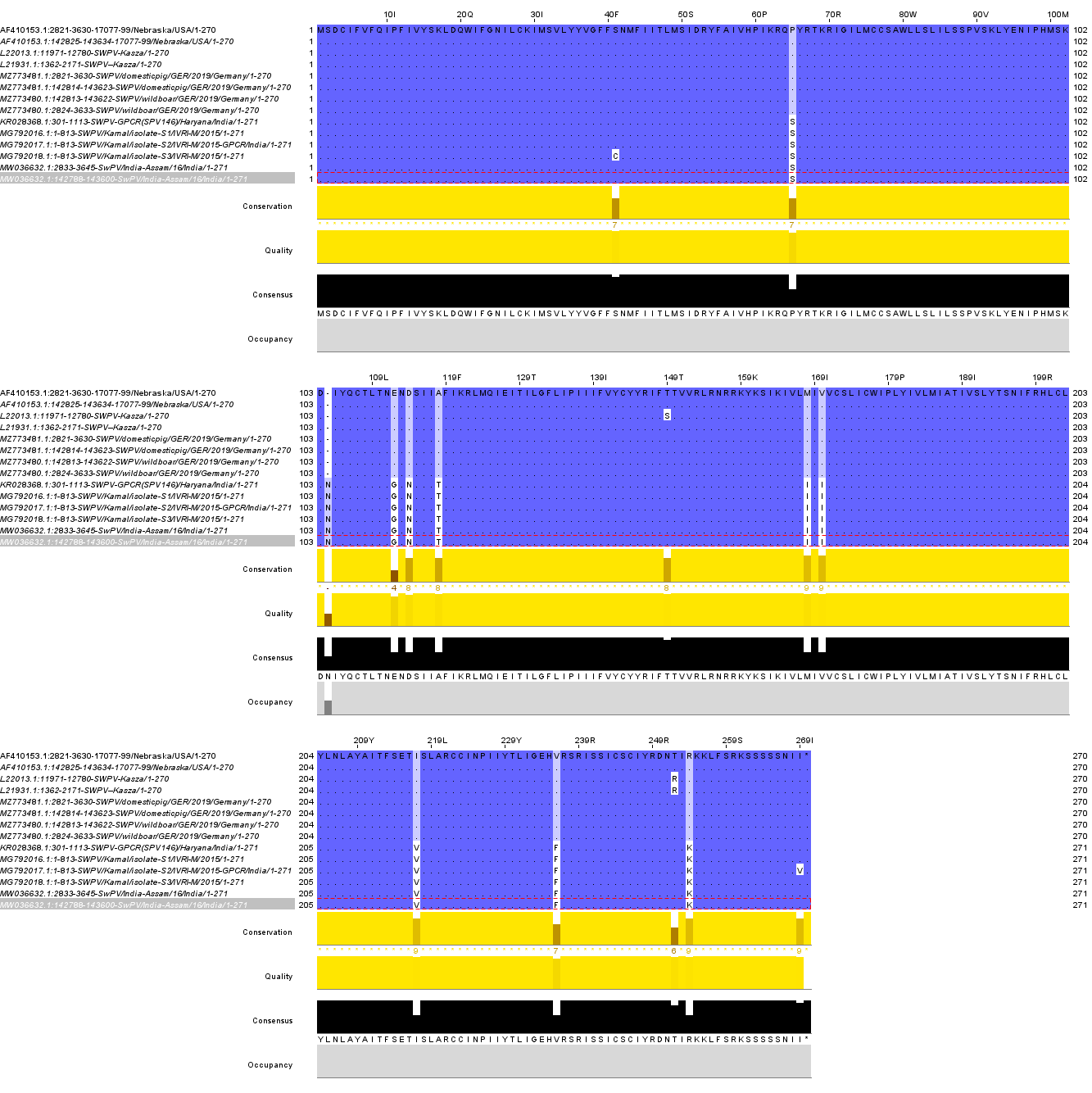


Fig. S4. Visualization of differential amino acid markers in ORF005/ORF146 of SWPV using Jalview 2.11.2.3. The conserved nts are dotted on the blue background and the differential markers are shown on a white background. A total of 10 differential markers could be observed between Indian lineage and European-North American lineage. Some isolate-specific markers could also be observed.


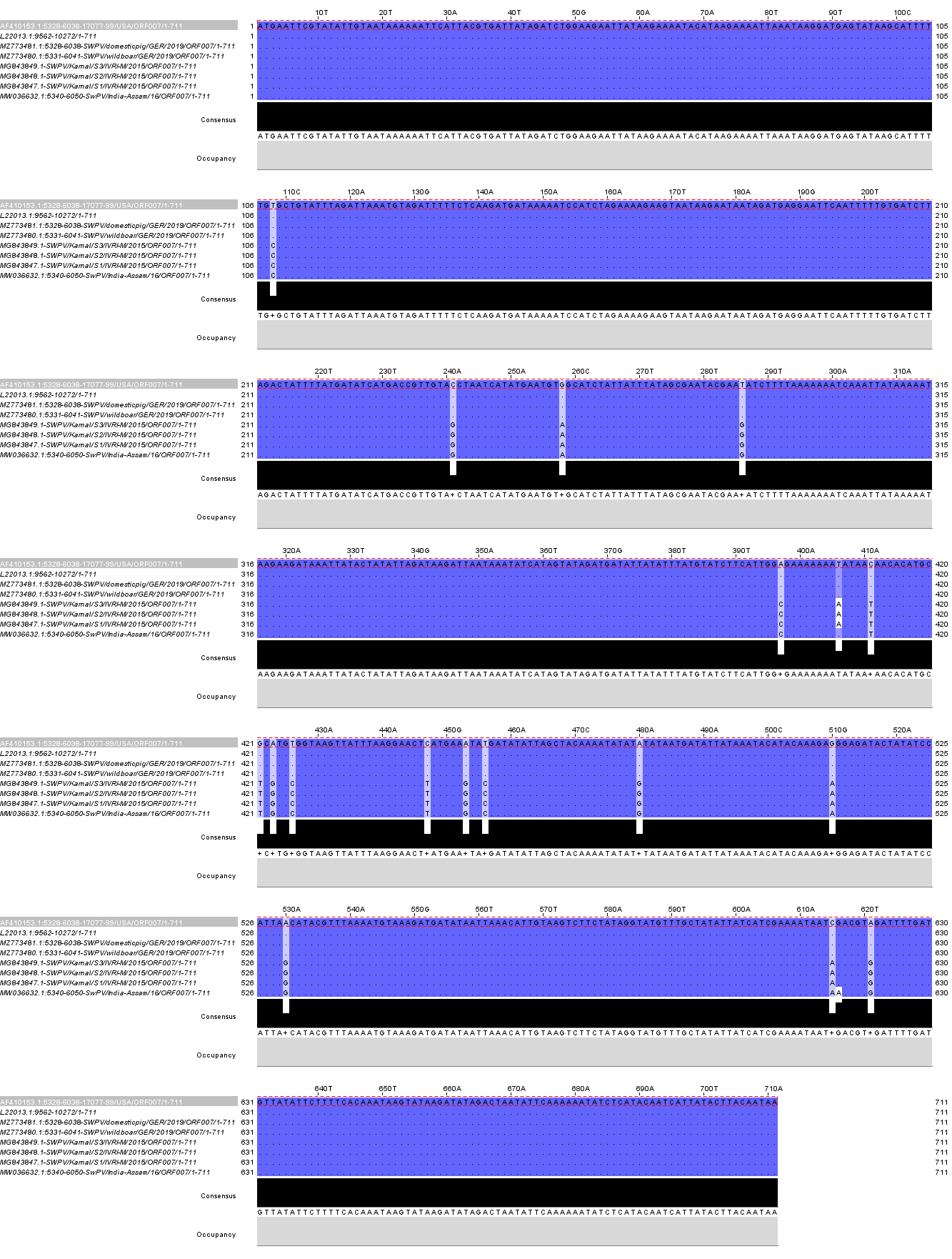


Fig. S5. Visualization of differential nucleotide markers (SNPs) in ORF007 of SWPV using Jalview 2.11.2.3. The conserved nts are dotted on the blue background and the differential markers are shown on a white background. A total of 17 differential markers could be observed between the Indian lineage and European-North American lineage. Some isolate-specific markers could also be observed.


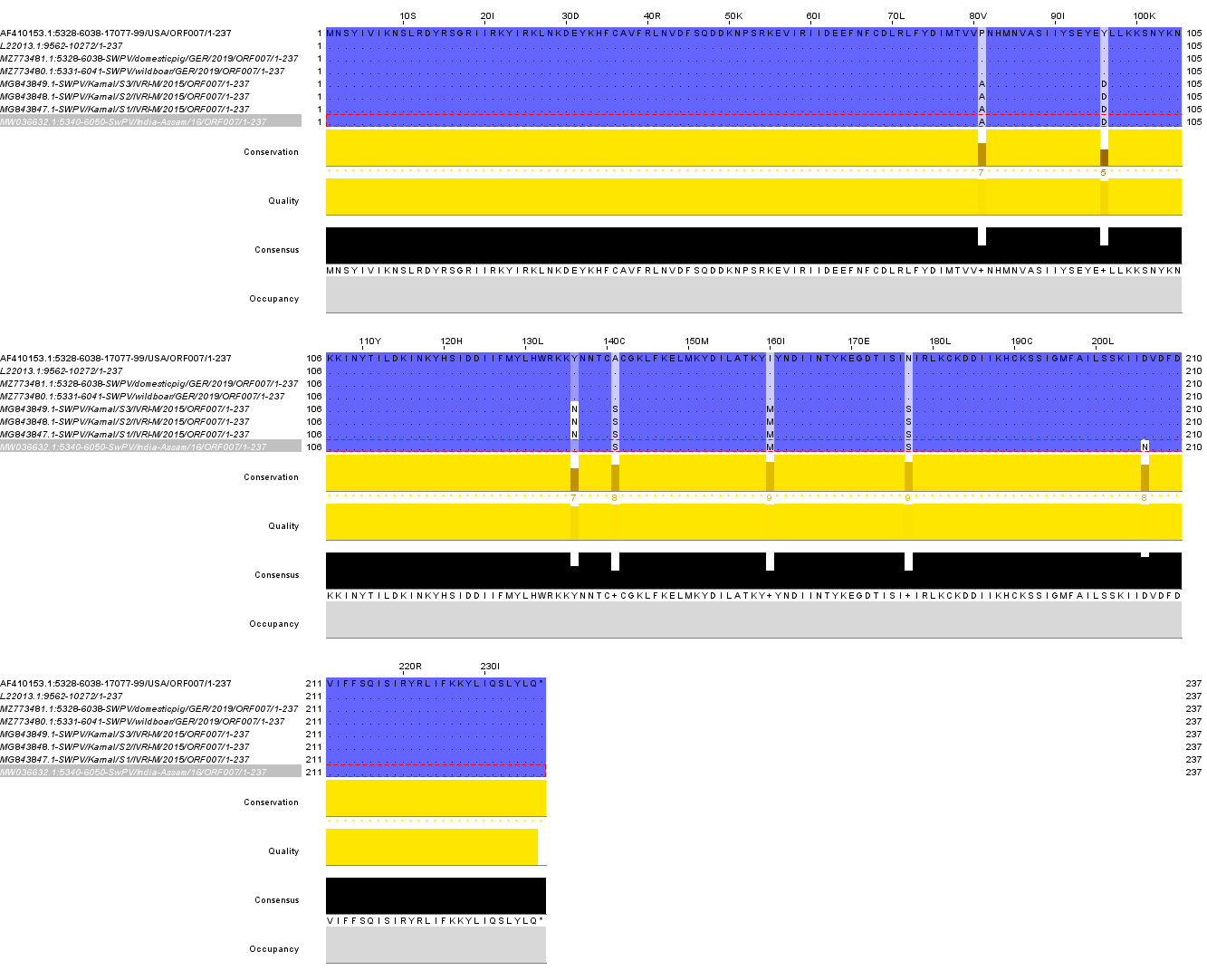


Fig. S6. Visualization of differential amino acid markers in ORF007 of SWPV using Jalview 2.11.2.3. The conserved nts are dotted on the blue background and the differential markers are shown on the white background. A total of 5 differential markers could be observed between Indian lineage and European-North American lineage. Some isolate-specific markers could also be observed.


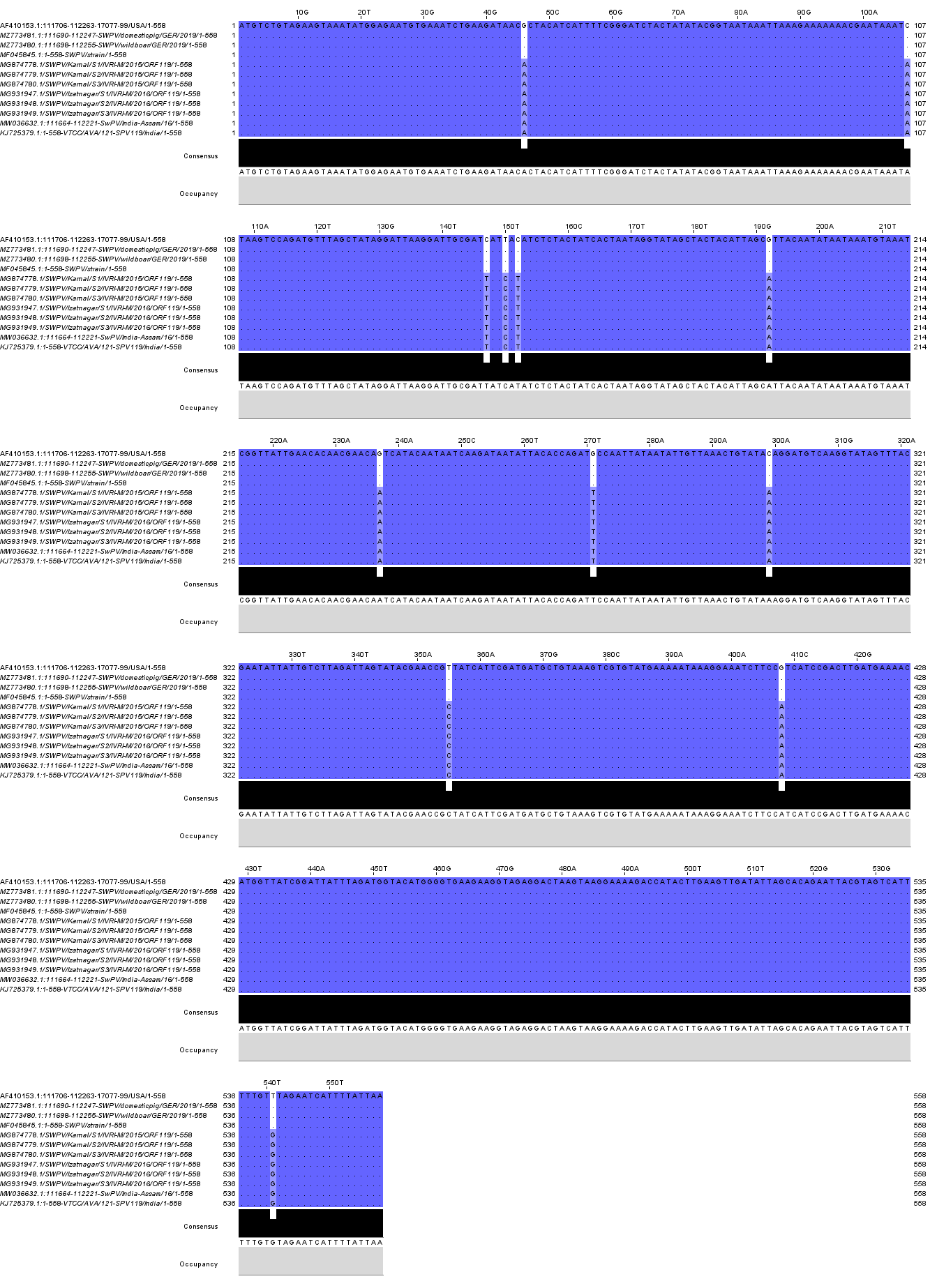


Fig. S7. Visualization of differential nucleotide markers (SNPs) in ORF119 of SWPV using Jalview 2.11.2.3. The conserved nts are dotted on the blue background and the differential markers are shown on a white background. A total of 12 differential markers could be observed between Indian lineage and European-North American lineage. Some isolate-specific markers could also be observed.

**
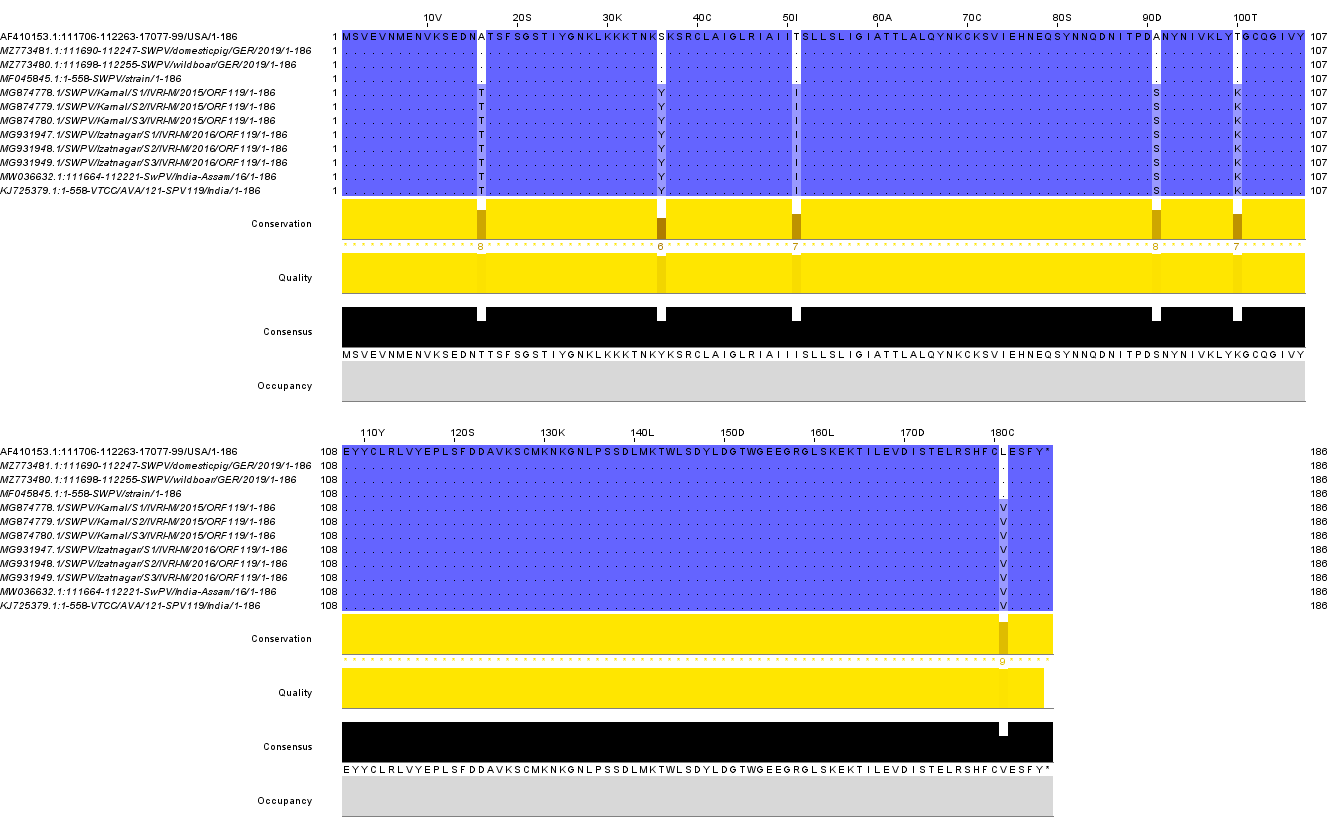
**

Fig. S8. Visualization of differential amino acid markers in ORF119 of SWPV using Jalview 2.11.2.3. The conserved nts are dotted on the blue background and the differential markers are shown on the white background. A total of 6 differential markers could be observed between Indian lineage and European-North American lineage. Some isolate-specific markers could also be observed.


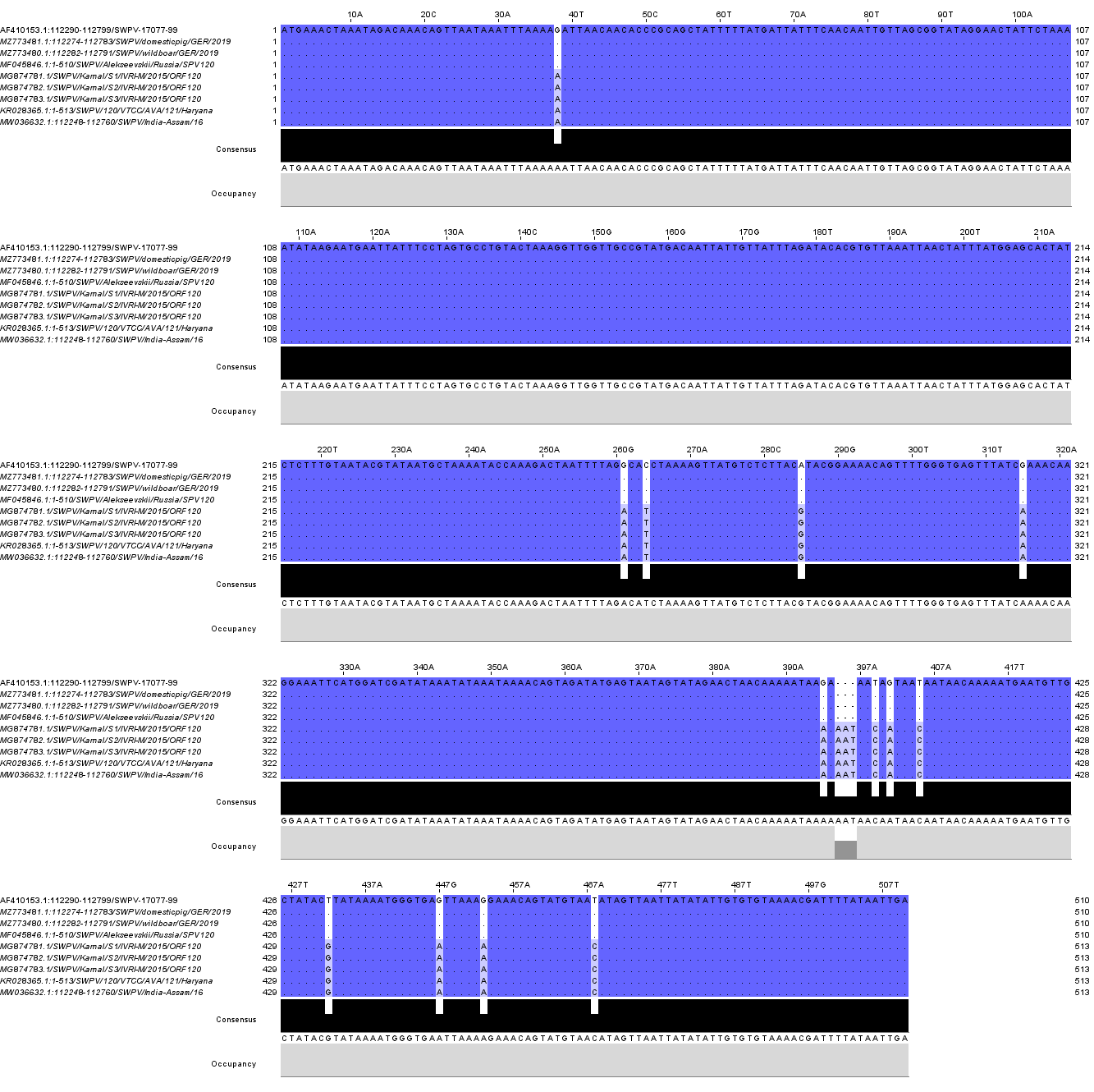


Fig. S9. Visualization of differential nucleotide markers (indels, SNPs) in ORF120 of SWPV using Jalview 2.11.2.3. The conserved nts are dotted on the blue background and the differential markers are shown on a white background. A total of 14 differential markers could be observed between the Indian lineage and European-North American lineage. Some isolate-specific markers could also be observed.


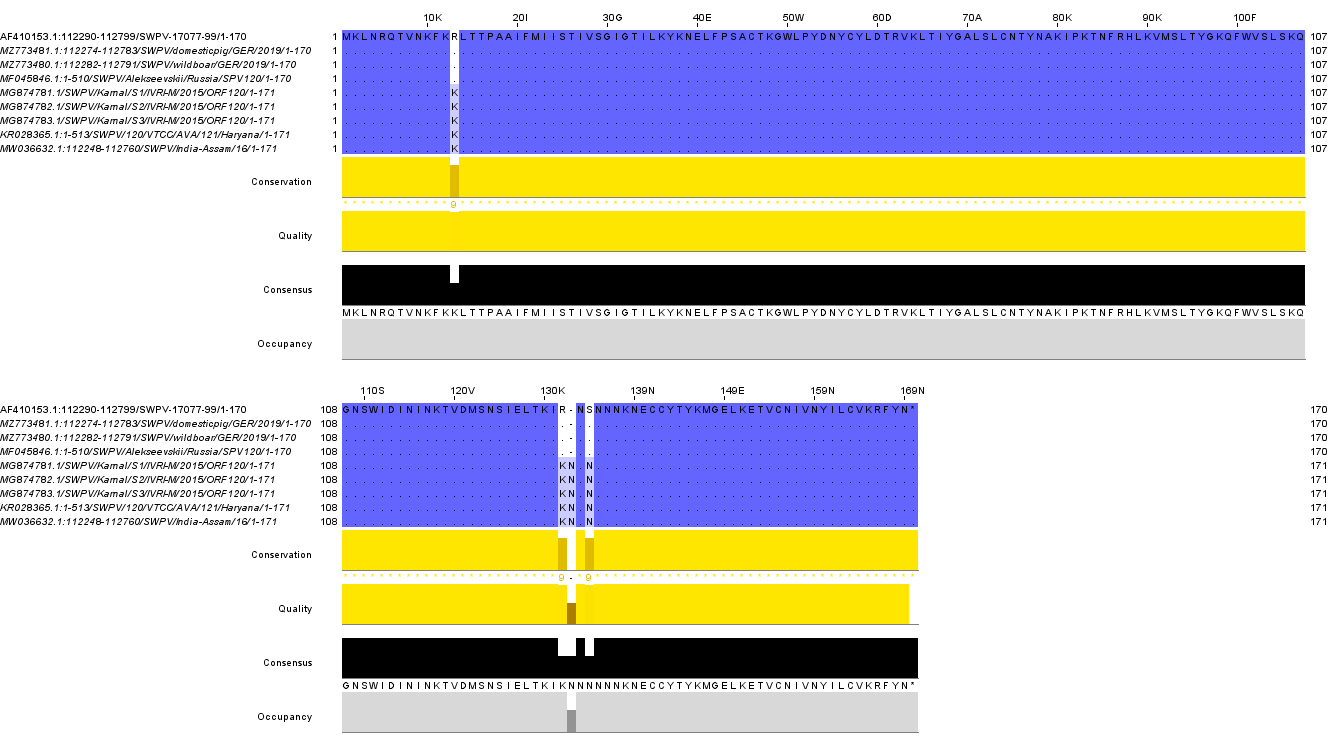


Fig. S10. Visualization of differential amino acid markers in ORF001/ORF150 of SWPV using Jalview 2.11.2.3. The conserved nts are dotted on the blue background and the differential markers are shown on the white background. A total of 4 differential markers could be observed between Indian lineage and

European-North American lineage. Some isolate-specific markers could also be observed.
